# Choroidal metastases from thymic carcinoma during pregnancy: Case Report

**DOI:** 10.1186/s12885-015-1968-4

**Published:** 2015-12-16

**Authors:** Sebastian P. Haen, Philipp Stroebel, Alexander Marx, Daniela Suesskind, Falko Fend, Ursula Reichmann, Hans-Georg Kopp, Lothar Kanz, Frank Mayer

**Affiliations:** 1 Medizinische Universitaetsklinik Tuebingen, Innere Medizin II fuer Onkologie, Haematologie, Immunologie, Rheumatologie und Pulmologie, Otfried Mueller Str. 10, D-72076 Tuebingen, Germany; 2 Interfakultaeres Institut fuer Zellbiologie, Abteilung Immunologie, Auf der Morgenstelle 15, D-72076 Tuebingen, Germany; 3 Pathologisches Institut, Universitaetsmedizin Mannheim, Theodor-Kutzer-Ufer 1-3, D-68167 Mannheim, Germany; 4 Institut fuer Pathologie, Universitaetsmedizin Goettingen, Robert-Koch-Str. 40, D-37075 Goettingen, Germany; 5 Departement fuer Augenheilkunde, Universitaetsklinikum Tuebingen, Schleichstr. 12, D-72076 Tuebingen, Germany; 6 Institut fuer Pathologie, Abteilung fuer Allgemeine Pathologie und Pathologische Anatomie, Universitaetsklinikum Tuebingen, Liebermeisterstr. 8, D-72076 Tuebingen, Germany; 7 Radioonkologische Klinik, Universitaetsklinikum Tuebingen, Hoppe-Seyler-Str. 3, D-72076 Tuebingen, Germany

**Keywords:** Thymic carcinoma, Uveal metastasis, Dermatomyositis

## Abstract

**Background:**

Rare sites of metastases, atypical symptoms and paraneoplastic syndromes are often neglected or misinterpreted, especially when they represent early symptoms of an underlying malignant disease. Hence, an interdisciplinary approach to these patients is essential to avoid tumor progression and metastatic spread in order to provide curative treatment options to the patients. We here report the case of a young woman presenting with visual loss which led to diagnosis of a thymic carcinoma.

**Case presentation:**

A 28-year old white woman presented with subacute loss of vision in the last trimester of her first pregnancy which was first interpreted as an exacerbation of a pre-existing dermatomyositis and treated with steroids. After failure of steroid therapy choroidal metastases from an undifferentiated thymic carcinoma were diagnosed. This also shed a new light on the dermatomyositis the patient had been suffering from for seven years possibly representing a paraneoplastic syndrome from the tumor. Despite aggressive chemotherapy, the patient died from progressive disease eight years after first onset of dermatomyositis and 14 months after initial diagnosis of the thymic carcinoma.

**Conclusions:**

Choroidal metastases from a thymic carcinoma have never been reported before but should be included into the differential diagnosis of choroidal masses.

## Background

Primary thymic carcinomas are rare tumors accounting for 7-25 % of all neoplasms of the thymus [1]. They are distinguished from the more frequent thymomas based on their different morphology and biology [2, 3]. Due to frequent invasion of pericardium or pleural space and metastatic spread, their prognosis is generally poor [4] with median survival times for patients with irresectable disease of 13 months [5]. Thymic carcinomas differ from thymomas also with respect to associated paraneoplastic autoimmune diseases. Thymomas are frequently associated with characteristic autoimmune phenomena like myasthenia gravis or pure red cell aplasia, which normally do not occur in thymic carcinomas. However, sporadic cases of polymyositis or hypercalcemia have been encountered in the latter [6–8].

Choroidal metastases are exceedingly rare. They can occasionally cause symptoms leading to the primary diagnosis of other tumors like lung and breast cancers, as well as melanoma [9, 10]. However, in most patients the underlying diseases are already diagnosed, and choroidal metastases represent late symptoms of the disease [10].

With the permission of the kin of the patient, we here report a case in which the presentation with visual loss resulted in the diagnosis of a thymic carcinoma which had not been found during earlier examinations.

## Case presentation

A 28-year old white woman presented with distinct deterioration of visual acuity during the last trimester of her first pregnancy. Her vision decreased to 20/40 in the left and 20/400 in the right eye. In the beginning, the visual loss occurred intermittently, but worsened over time. In addition, the patient complained about flickering. During the worst episodes she could only see contours with a vague discrimination of light. Simultaneously, symptoms of a dermatomyositis, which had been diagnosed and successfully treated several years earlier, reappeared. At that time, the diagnosis of dermatomyositis had been confirmed by skin biopsy. An extensive workup to rule out an underlying malignancy had then not revealed any suspicious results. However, a CT scan had not been performed. The patient subsequently had received several immunosuppressive treatments including azathioprine, methotrexate and adalimumab, as well as immunoglobulins and steroids resulting in a long-lasting remission of the dermatomyositis.

**Fig. 1:**
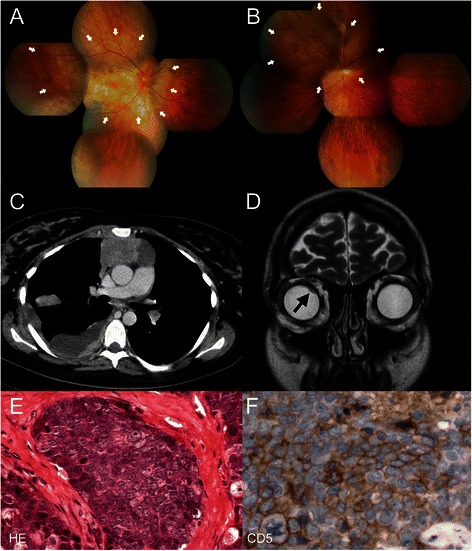
Diagnostic evaluation. **a** and **b** Composite color fundus photographs of both eyes showing the amelanotic choroidal lesions (arrows) at the posterior pole of the right eye (**a**) and predominantly nasal superior to the optic disc in the left eye (**b**). **c** and **d** Radiographic imaging. CT scan of the chest (**c**). Note the large mediastinal mass with pleural spreading. MRI scan of the orbits (**d**). The arrow marks the choroidal lesion. **e** and **f** Histological appearance of the undifferentiated thymic carcinoma (400-fold magnification). The H&E stain (**e**) shows compact nests of undifferentiated epithelial cells with narrow cytoplasm without evidence of keratinization, large vesicular nuclei and high mitotic rate, separated by broad collagen bands (immunostaining for CD5). The tumor cells show a strong expression of CD5 (**f**) characteristic for an undifferentiated thymic carcinoma

The decrease in visual acuity occurred isochronal with another exacerbation of skin symptoms and was therefore initially interpreted as a manifestation of the reappearing dermatomyositis and treated with steroids since also initial ophthalmologic workup did not reveal pathological results. After failure of immunosuppressive therapy, repeated ophthalmologic examination revealed an amelanotic choroidal mass at the posterior pole including the peripapillary region and a second choroidal lesion superior to the optic disc in the right eye. Also, in the left eye another amelanotic choroidal tumor situated predominantly nasal superior to the optic disc was detected. In both eyes, an inferior exsudative retinal detachment was seen (Fig. 1a and b). Finally, a large tumor mass in the mediastinum, pleural thickening and pulmonary nodules as well as a mediastinal lymphadenopathy were detected in MRI scans. CT scans were not performed because of the actual pregnancy. After delivery of a healthy boy at the 37th + 2 week of pregnancy through Caesarean section, a lung and pleural biopsy was performed by lateral thoracotomy and the patient was referred to our center for further treatment.

The clinical examination showed a 28-year old woman in proper general condition. Her skin was reddish and thickened with distinct flaking. The examination of the heart, lungs and abdomen did not reveal any pathologic results. Decreased visual acuity was noted in both eyes. Additionally, the patient complained about paraesthesia with tickling in both feet. Other neurological symptoms were not detectable.

Laboratory testing revealed a slightly increased creatine kinase (193 U/l, normal up to 170 U/l), an elevated C-reactive protein (2.77 mg/dl, normal up to 0.5 mg/dl), anemia with haemoglobin of 9.8 g/dl, elevated uric acid levels of 6.5 mg/dl (reference range 2.4 to 5.7 mg/dl) and a significantly increased lactate dehydrogenase of 959 U/l (normal up to 250 U/l) as well as thrombocytosis of 789 G/μl (normal up to 450 G/μl).

Whole body CT and MRI scans showed metastases to the choroids, pleura and regional lymph nodes (Fig. 1b and c). The lung biopsy revealed a poorly differentiated carcinoma with strong expression of cytokeratins 5/6, CD5 and CD117 and absence of neuroendocrine markers (CD56; chromogranin, synaptophysin), a constellation highly specific for thymic carcinomas (Fig. 1e and f). A lymphoepithelioma-like thymic carcinoma was ruled out by negative EBV in situ hybridization. In addition, a thymic carcinoma with t (15;19) translocation was likewise ruled out by specific real-time polymerase chain reaction (RT-PCR), which failed to demonstrate a BRD4-NUT gene fusion product. Thus a final diagnosis of an undifferentiated thymic carcinoma was established. The tumor at initial diagnosis presented in stage IVb (T4, N2 (hilar lymph nodes), M1b (pleura, lung, choroid)) [11, 12].

Thymic carcinomas are often moderately differentiated squamous cell carcinomas histologically resembling the appearance of squamous cell carcinomas elsewhere in the body, e.g. the lung [13, 14]. In our patient, one potential differential diagnosis was the lymphocyte poor EBV-associated lymphoepithelioma-like carcinoma, an aggressive tumor with a poor prognosis [15, 16]. However, EBV association was ruled out by in situ hybridisation. Another differential diagnosis in young adults with a rapidly progressive carcinoma is the so-called thymic carcinoma with t (15; 19) translocation, which is associated with a fatal prognosis, including rapid local invasion and systemic dissemination [17–20]. In our case, this diagnosis could also be ruled out, since the BRD4-NUT fusion gene transcript resulting from this chromosomal translocation could not be detected. Hence, a final diagnosis of a high grade, poorly differentiated thymic carcinoma was established by exclusion of other possible subtypes of primary thymic carcinomas.

Classical metastatic spread patterns of thymic carcinoma comprise regional anterior perithymic, deep intrathoracic and cervical lymph nodes, as well as the pleura, the pericardium and the lung [12]. Extrathoracic organ metastases mainly affect the liver and the kidney, but may also involve the bones [4, 21–24]. Also one case of orbit metastases from a neuroendocrine thymic carcinoma was reported [25].

To our knowledge, our patient represents the first case of choroidal metastases from thymic carcinoma. Although choroidal metastases are generally rare (e.g. about 5-10 % in breast and lung carcinoma patients, respectively [26, 27]), the choroid represents the most common ocular site for metastatic disease (up to 88 % of secondary ocular tumors) which is due to hematogenenous dissemination into abundant choroidal vasculature [9, 28, 29]. The most common primary tumors presenting with choroidal metastases are breast (40-53 %) [9, 26, 30–32] and lung carcinoma (20-29 %) [9, 30, 33] but may also include tumors (2-4 % each) from the gastrointestinal tract, the kidney, the prostate and the skin [9, 30, 34–39], as well as carcinoid tumors of different localisation including one reported case of a thymic carcinoid [40]. Evidence for choroidal metastases from other tumors has emerged in recents years. These observations are mainly limited to single case reports and comprise metastases from malignancies of the thyroid [29, 41–45], the urogenital tract [46–49], the pancreas [50, 51], salivary glands [52, 53], and the chorion [54], as well as sarcomas [55, 56].

In our patient, fractioned radiation therapy of the orbits with a cumulative dose of 30 Gy (10 × 3 Gy) using opposite, coplanar and coaxial fields in an isocentric adjustment with 6 MV photons was performed and resulted in regression of the choroidal metastases and an improvement of visual acuity from 20/400 to 20/40 in the right eye and from 20/40 to 20/16 in the left eye resulting in reconstitution of reading ability. Afterwards, chemotherapy with Cisplatin (75 mg/m^2^) and Paclitaxel (175 mg/m^2^) was initiated as a t (15; 19)-positive carcinoma was still assumed at that time. Due to the rapid clinical response, this treatment was continued for a total of six cycles after receiving the final pathology report. After four cycles, a good partial response was observed in CT scan. However, progressive disease was evident in the staging after six cycles. The regime was then changed to a modified PAC-scheme (Carboplatin AUC5 instead of cisplatin because of a suspected paclitaxel-induced peripheral polyneuropathy CTC grade 1, Doxorubicin 50 mg/m^2^ and Cyclophosphamide 500 mg/m^2^). Nevertheless, the tumor progressed further, and the patient died of progressive disease in respiratory insufficiency eight years after first manifestation of the dermatomyositis and fourteen months after primary diagnosis. The median overall survival of patients with undifferentiated thymic carcinoma has been reported to be about six months [57].

Retrospectively, the pre-existing dermatomyositis might have represented the first symptom of an at that time clinically occult tumor. The association between the epithelial thymic carcinoma and the dermatomyositis in this case could not be totally proven but such an association has been reported in some cases of thymomas [58], but appears to be extremely rare in thymic carcinomas with only two reports in the literature [59, 60].

## Conclusions

Our case demonstrates that thymic carcinomas should be included into the differential diagnosis in cases with choroidal metastases and with dermatomyositis and no detectable tumor, especially in young adults. To our knowledge, this is the first case of a thymic carcinoma with highly aggressive and fatal course and atypical clinical presentation with choroidal metastases.

## Consent

Written informed consent was obtained from the patient’s relatives for publication of this case report and the accompanying images. A copy of the written informed consent is available for review by the Editor-in-Chief upon request.

## Abbreviations

BRD: bromodomain-containing protein
CD: cluster of differentiation
CT: computed tomography
EBV: epstein barr virus
HE: hematoxylin eosin
MRI: magnetic resonance imaging
MV: megavolt
NUT: nuclear protein in testis
PAC: platinum adriamycin cyclophosphamide
RT-PCR: real-time polymerase chain reaction
